# Idiopathic Sudden Sensorineural Hearing Loss in Different Ages: Prognosis of Patients With Initial Total Hearing Loss

**DOI:** 10.3389/fpsyg.2022.818967

**Published:** 2022-03-24

**Authors:** Wenping Xiong, Qinglei Dai, Yingjun Wang, Zhiqiang Hou, Kunpeng Lu, Xiao Sun, Fujia Duan, Haibo Wang, Daogong Zhang, Mingming Wang

**Affiliations:** Department of Otolaryngology-Head and Neck Surgery, Shandong Provincial ENT Hospital, Cheeloo College of Medicine, Shandong University, Jinan, China

**Keywords:** idiopathic sudden sensorineural hearing loss, aging, vertigo, prognosis, total hearing loss

## Abstract

**Objective:**

This study aimed to analyze the hearing improvement and prognosis factors of idiopathic sudden sensorineural hearing loss (ISSNHL) in different ages with initial total hearing loss.

**Methods:**

We reviewed the medical records of 5,711 hospitalized patients with ISSNHL from 2016 to 2021 in our center. All of the patients had been treated with uniform combination drug therapy. After excluding the patients with initial partial hearing loss and those diagnosed with clear etiology, 188 patients were enrolled in this study and divided into six age groups (18–30, 31–40, 41–50, 51–60, 61–70, ≥ 71 years). In all groups, decreases in pure-tone average (PTA) 1 month posttreatment, effective rate, and clinical characteristics (vertigo, tinnitus, hospital stay, comorbidity, and inner ear magnetic resonance imaging) were analyzed.

**Results:**

Among the 188 enrolled patients, 86% had vertigo. Complete recovery was seen in 0.5% of patients, and marked recovery was seen in 43% of patients. The mean 1 month posttreatment PTAs were as follows: 18–30 years: 80 ± 7.5 dB; 31–40 years: 100 ± 9.0 dB; 41–50 years: 99 ± 8.3 dB; 51–60 years: 101 ± 8.6 dB; 61–70 years: 96 ± 9.6 dB; and ≥ 71 years: 88 ± 13.0 dB. Compared with the other groups, the 18–30- years group showed better recovery of hearing threshold in five frequencies (0.25, 0.5, 1, 2, and 4 kHz, respectively, at octave or semioctave frequencies under air conduction), and the recovery of hearing threshold at 0.25 and 0.5 Hz was better than the recovery at 1, 2, and 4 kHz. According to the results of the chi-square test statistical analysis, vertigo and comorbidities were associated with a poor prognosis of ISSNHL.

**Conclusion:**

In summary, the treatment outcomes of patients with ISSNHL with initial total hearing loss were poor. There was a significant age-related difference with respect to marked recovery 1 month posttreatment, and the 18–30- years group showed better recovery than the other age groups.

## Introduction

Idiopathic sudden sensorineural hearing loss (ISSNHL) is an otologic emergency. Hearing loss and vertigo are the main complaints associated with it. ISSNHL has been defined as a sensorineural hearing loss > 30 dB over at least 3 contiguous audiometric frequencies within 3 days or less with no identifiable causes (Chandrasekhar et al., 2019). Despite the lack of accurate incidence reports in China, our patient records show that the incidence has increased in the recent years. In the United States, the annual incidence of ISSNHL was 27 per 1,00,000 patients between 2006 and 2007. The incidence increased with age, ranging from 11 per 1,00,000 for patients younger than 18 years to 77 per 1,00,000 for patients aged 65 years and older. In addition, there was a slight male predominance, with a male-to-female ratio of 1.07:1. This was more pronounced in patients aged 65 years and older, with a ratio of 1.30:1 (Alexander and Harris, 2013).

Idiopathic sudden sensorineural hearing loss is a heterogeneous disease in terms of clinical symptoms (with/without vertigo, tinnitus, and fullness), causes (viral infection, immunologic causes, and vascular), the severity of hearing loss (mild, moderate, severe, and profound), and prognosis. Profound ISSNHL has a particularly poor prognosis, regardless of the treatment type and hospital stay. Although numerous studies concerning severe to profound ISSNHL have been published (Hong et al., 2012; Thomas et al., 2018; Wei et al., 2019; Lv et al., 2022), most did not completely separate total and subtotal hearing loss based on the initial pure-tone audiometry. Few articles have focused on total hearing loss, especially in patients with severe vertigo. Furthermore, most studies on total ISSNHL included small patient groups. Most patients with ISSNHL with initial total hearing loss did not respond to the therapy prior to hospitalization. This adversely affected the patients emotionally as the clinical course of the recovery after ISSNHL with initial total hearing loss is not known.

Therefore, we evaluated hearing improvement after treatment in different-age patients who presented with total hearing loss. We paid special attention to the improvement in the pure-tone average (PTA) 1 month posttreatment.

## Materials and Methods

### Subjects

This retrospective study included patients with initial total hearing loss due to ISSNHL hospitalized at the Department of Otologic Center, Shandong Provincial ENT Hospital between January 2016 and August 2021. Some patients were referred from other hospitals for further treatment in our department because of poor recovery. This study was approved by the hospital ethics review board and performed in accordance with the Declaration of Helsinki.

The patient inclusion criteria were as follows: (1) age ≥ 18 years, (2) diagnosis of unilateral ISSNHL, where the initial pure-tone audiometry produced no responses (0.25, 0.5, 1, 2, 4, and 8 kHz air conduction thresholds exceeded the audiometer’s maximum:105, 120,120,120,120,and 100 dBHL), (3) follow-up ≥ 1 month, and (4) treatment for ≥ 14 days.

The exclusion criteria were as follows: (1) bilateral sudden hearing loss, (2) age < 18 years, (3) history of hearing loss, (4) middle ear disease, and (5) other distinct diagnoses related to known causes of rapid hearing loss, such as injury, labyrinthine fistula, cytomegalovirus infection, Hunt syndrome, psychogenic deafness, Meniere’s disease, congenital and genetic hearing loss (large vestibular aqueduct), systemic immunological disease, vestibular Schwannoma, and noise-induced hearing loss.

### Treatment

All patients were hospitalized for ≥ 14 days (including those referred from other hospitals). The combination therapy administered was as follows: (1) intravenous drip dexamethasone: 10 mg/day consecutive for 3 days and 5 mg/day consecutive for 4 days; (2) postauricular injection of methylprednisolone (40 mg) once every 2 days until discharge; (3) Ginkgo biloba extract: 105 mg/day intravenous drip for 14 days; and (4) monosialoganglioside 80 mg/day intravenous drip for 7 days. After discharge, the patients received oral mecobalamin and Ginkgo tablets.

### Audiological Examinations and Follow-Up

The patients gave a full medical history and underwent a comprehensive audiological evaluation that includes otoscopic examination, pure-tone audiometry (Grason Stadler, AudioStar61, United States), tympanometry (Grason Stadler Tympstar), distortion products otoacoustic emissions (DPOAEs, Intelligent Hearing Systems Smart EP), auditory brainstem responses (ABRs, Intelligent Hearing Systems Smart EP), and other systematic examinations for hearing impairment during hospitalization. Inner ear magnetic resonance imaging (MRI) was performed on a 3T clinical scanner (Discovery 750 w, GE Healthcare, Waukesha, United States). A 3D-FLAIR sequence and a 3D-T2WI sequence were employed. The following variables that might influence improvement were recorded: age, sex, affected side, onset season, time between ISSSHL onset and the start of treatment, hospital stay, vertigo, tinnitus, MRI of the inner ear, arterial hypertension, diabetes, dyslipidemia, and thyroid disorders.

Pure-tone averages (at 0.25, 0.5, 1, 2, and 4 kHz, respectively) at octave or semioctave frequencies under air conduction and 0.25–4 kHz under bone conduction were measured two times per week during treatment and once every 2 weeks after the completion of treatment. The average air-conduction PTAs were obtained through the thresholds at frequencies of 0.25, 0.5, 1, 2, and 4 kHz, respectively, following the “Kanzaki criteria” (Kanzaki, 1999; Table 1). When the hearing thresholds were undetectable, fixed values were applied for calculations as follows: 110–125–125–125–125 (0.25, 0.5, 1, 2, and 4 kHz, respectively, maximum outputs of our hospital audiometer were 105–115–115–115–115 dB, plus 5, respectively) (Fontenot et al., 2019). Hearing gain was classified into four grades according to the PTA gain (Kanzaki, 1999). Accordingly, we classified patients into the recovery (i.e., complete and marked recovery) and no recovery groups (i.e., slight and no recovery). The patients were also divided into six age groups (18–30, 31–40, 41–50, 51–60, 61–70, and ≥ 71 years). In addition, the patients’ hearing thresholds were tested every 2 weeks after the completion of treatment until 3 months after onset.

**TABLE 1:** Criteria of hearing recovery (according to Kanzaki’ criteria).

I.	Complete recovery	Hearing thresholds recovered to less than 25 dB, or to the same level of the other ear
II.	Marked recovery	Hearing gain average of the 5 frequencies (0.25, 0.5, 1, 2, and 4 kHz) ≥ 30 dB
III.	Slight recovery	Hearing gain average of the 5 frequencies (0.25, 0.5, 1, 2, and 4 kHz) <30 dB
IV.	No recovery Hearing	Gain average of the 5 frequencies (0.25, 0.5, 1, 2, and 4 kHz) <10 dB

### Statistical Analysis

Statistical analyses were performed using SPSS statistical software (version 26). The Fisher’s exact test and the chi-square test were applied to categorical data, variance analysis to quantitative data, and the Mann–Whitney and Kruskal–Wallis tests to quantitative and ordinal data. Chi-square test was conducted to assess the effects of different variables on hearing prognosis. Statistical significance was determined at a confidence level of *p* < 0.05.

## Results

**FIGURE 1:**
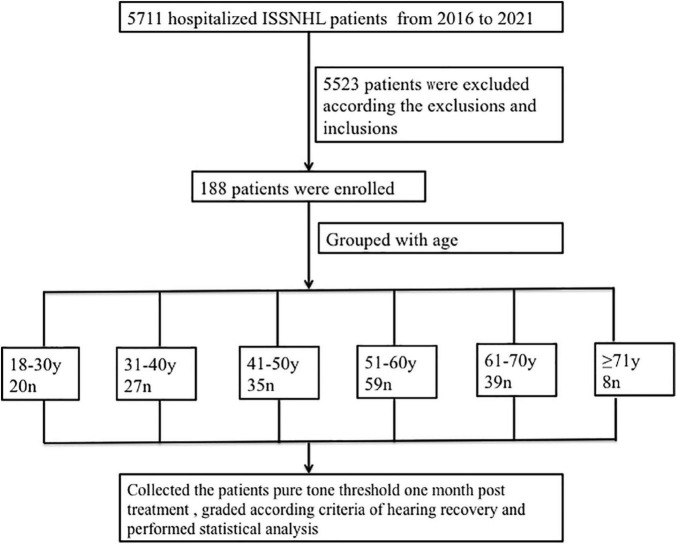
Research flow diagram. ISSNHL, idiopathic sudden sensorineural hearing loss; n, number; y, year.

From the 5,711 screened medical records, 5,523 patients were excluded for the following reasons: (1) the initial hearing loss was not total (*n* = 5,365), (2) bilateral ISSNL (*n* = 8), (3) age < 18 years (*n* = 35), (4) previous hearing loss (*n* = 3), (5) middle ear disease (*n* = 3), (6) injury (*n* = 2), (7) cytomegalovirus infection (*n* = 1), (8) Hunt syndrome (*n* = 1), (9) psychogenic deafness (*n* = 3), (11) large vestibular aqueduct (*n* = 3), (12) vestibular Schwannoma (*n* = 2), (13) lack of 30-day follow-up after treatment (*n* = 94), and (14) inner ear malformation (*n* = 3). Finally, 188 patients were enrolled and divided into 6 age groups (18–30 years: *n* = 20; 31–40 years : *n* = 27; 41–50 years: *n* = 35; 51–60 years: *n* = 59; 61–70 years: *n* = 39; ≥ 71 years: *n* = 8) (Figure 1). All patients’ baseline audiograms were identical (total hearing loss).

### Clinical Characteristics

Patient demographics are summarized in Table 2. The mean age of the 188 enrolled patients was 50.15 ± 13.46 years (range: 18–80 years). There were 107 women and 81 men, with equal numbers of left and right affected ears (*n* = 94). There were no significant differences in side of involvement, sex, time from onset to treatment (≤ 7 days is not listed in Table 2), results of inner ear MRI (hemorrhage/no hemorrhage: 105/83 not listed in Table 2), and cause of onset.

**TABLE 2:** Clinical profiles of patients and hearing recovery with respect to clinical predictor factor.

Parameter	Sex		Side		Vertigo	Tinnitus	Hospital stay		Comorbidity (Hypertension, Diabetes, Dyslipidemia, Thyroid disorder)	Hearing improvement			
						
Age	Female	Male	Right	Left			≤ 14	>14		I	II	III	IV
18–30 years	11	9	10	10	16	18	5	15	2	1	16	1	2
31–40 years	15	12	13	14	27	25	14	13	7	0	8	11	8
41–50 years	20	15	18	17	30	31	22	13	7	0	16	4	15
51–60 years	31	28	29	30	50	52	37	22	41	0	19	14	26
61–70 years	25	14	22	17	35	36	22	17	26	0	18	9	12
≥71 years	5	3	2	6	4	6	4	4	6	0	4	0	4
*P*-value	0.921		0.473		**0.014**	0.752	0.081		**0.000**	0.133	**0.006**	0.016	0.078

*The bold values mean statistic differences.*

### Hearing Outcomes 1 Month After Treatment

**FIGURE 2:**
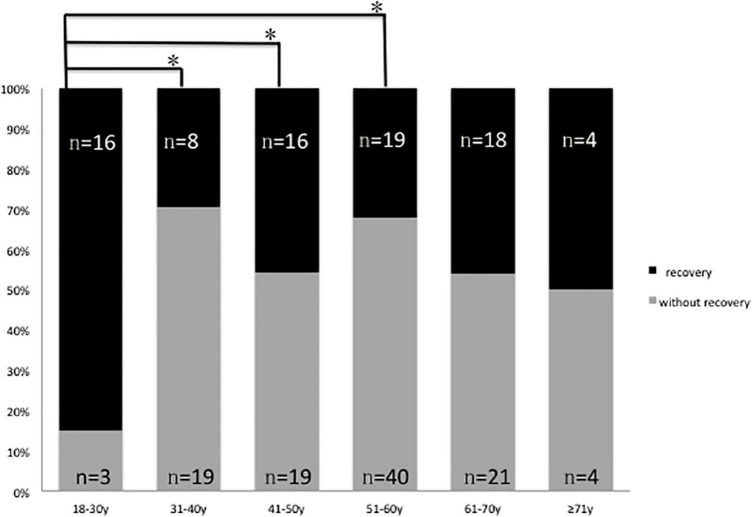
Graphical presentation of the percentage of patients with (II, marked recovery) and without recovery (III + IV slight recovery + no recovery) in six groups, black: Number of patients with marked recovery (II) 1 month posttreatment; gray: Number of patients with slight recovery and no recovery (III + IV) 1 month posttreatment *p 0.05.

In this study, only one 22-year-old male patient recovered completely (I). The mean 1 month posttreatment PTAs in the six groups were as follows: 18–30 years: 80 ± 7.5 dB; 31–40 years: 100 ± 9.0 dB; 41–50 years: 99 ± 8.3 dB; 51–60 years: 101 ± 8.6 dB; 61–70 years: 96 ± 9.6 dB; and ≥ 71 years: 88 ± 13.0 dB. The Kruskal–Wallis test showed a significant difference among all the six age groups (*p* = 0.009). When comparing the marked recovery (II) status in all groups, there were significant differences between the following groups (Table 3 and Figure 2): 18–30- years (16 of 20 patients, 80%) and 31–40-year groups (8 of 27 patients, 30%; *p* = 0.001), 18–30 and 41–50-year groups (16 of 35 patients, 46%; *p* = 0.013), and 18–30 and 51–60-years groups (19 of 59 patients, 32%; *p* = 0.000). The chi-square test was used to compare the recovery (complete and marked recovery, I + II) and non-recovery groups (slight and no recovery, III + IV) among the six age groups. There was a significant difference in the 18–30-years (17 of 20 patients, 85%; *p* = 0.002) and 31–40-years (8 of 27 patients, 30%; *p* = 0.000) groups and the 18–30 and 51–60-years groups (19 of 59 patients, 32%; *p* = 0.000). However, there was no significant difference in the other groups. There were no statistical differences between the 18–59 and ≥ 60 y in I, II, III, and IV or in I + II, III + IV (*p* = 0.953, *p* = 0.844; Table 4).

**TABLE 3:** Distribution of PTA recovery 1 month posttreatment (six groups).

Hearing recovery	18–30 years	31–40 years	41–50 years	51–60 years	61–70 years	≥ 71 years
I	1	0	0	0	0	0
II	16	8	16	19	18	4
III	1	11	4	14	9	0
IV	2	8	15	26	12	4
I + II	17	8	16	19	18	4
III + IV	3	19	19	40	21	4

**TABLE 4:** Distribution of PTA after treatment of 1 month (two groups).

Age groups	I	II	III	IV	I + II	III + IV
18–59 years	1	58	28	48	59 (44%)	76 (56%)
≥60 years	0	24	10	19	24 (45%)	29 (55%)

**FIGURE 3:**
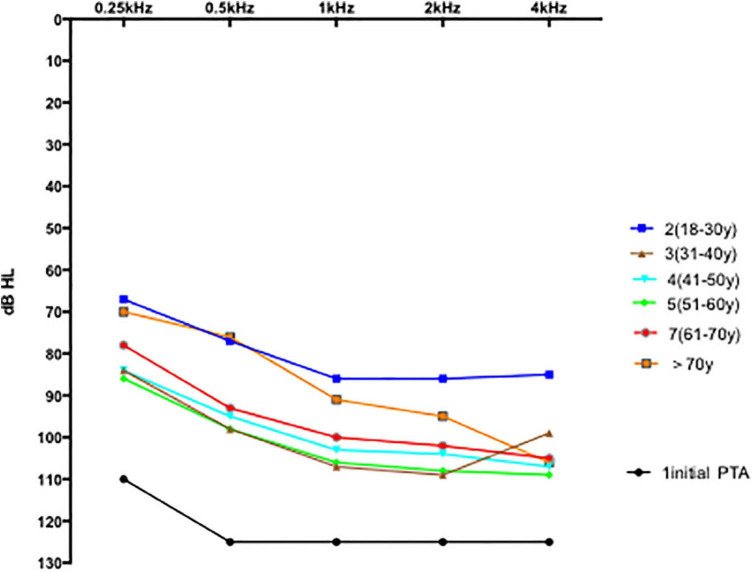
PTA in six groups 1 month posttreatment.

Comparisons of hearing gain at five frequencies (Table 5) revealed significant differences (*p* = 0.001); the hearing gain in the 18–30-years group was higher than in the other groups (18–30 vs. 31–40 years, *p* = 0.004; 18–30 vs. 41–50 y, *p* = 0.008; 18–30 vs. 51–60 years, *p* = 0.001). The recovery of hearing at 0.25 and 0.5 Hz was better than the recovery at 1, 2, and 4 kHz. The mean PTA in all groups 1 month posttreatment is shown in Figure 3. The 1 month posttreatment PTA in all groups was still severe to profound.

**TABLE 5:** Mean hearing gain values at each octave frequency 1 month posttreatment in the six groups.

Frequency (Average ± SD)	250 Hz	500 Hz	1 kHz	2 kHz	4 kHz
18–30 years	48.54 ± 29.25	50.75 ± 25.61	41.50 ± 20.14	41.50 ± 22.89	41.75 ± 24.08
31–40 years	30.00 ± 23.16	30.56 ± 22.59	20.74 ± 19.69	18.15 ± 17.60	13.33 ± 18.96
41–50 years	26.29 ± 25.48	30.14 ± 30.04	23.14 ± 23.64	21.14 ± 24.23	18.71 ± 24.92
51–60 years	24.32 ± 26.56	26.95 ± 26.08	18.73 ± 22.33	16.61 ± 22.33	16.19 ± 22.33
61–70 years	34.49 ± 30.60	34.74 ± 29.09	29.49 ± 28.00	27.69 ± 26.23	24.87 ± 26.44
≥71 years	28.75 ± 34.10	32.50 ± 39.37	25.63 ± 31.90	22.50 ± 26.73	13.13 ± 18.89

### Prognostic Factors

Vertigo (*p* = 0.014) and comorbidity (*p* = 0.000) were scored as prognostic factors (Table 2).

## Discussion

The incidence of ISSNHL has increased in the recent years and its etiology is still unknown. The pathophysiology of the changes in the cochlea in a sudden hearing loss *in vivo* is unknown. Overall, ISSNHL is a challenging condition. Although 65% of patients with ISSNHL can spontaneously recover functional hearing levels without treatment (Mattox and Simmons, 1977), patients with an initial hearing level > 100 dB reported unsatisfactory results (8.1–9.1%) (Kim et al., 2020). This study aimed to analyze hearing recovery at 1 month posttreatment in 188 patients with a sudden and total hearing loss.

Among all hospitalized patients from 2016 to 2021, 6% (346/5,711), including those aged less than18 years, presented with total initial hearing loss. We excluded 35 patients aged less than 18 years because the recovery rate of ISSNHL in pediatric patients is higher than in adults (Chung et al., 2015), and two more were excluded due to psychogenic deafness (ABR and DPOAE). Clinically, vestibular Schwannoma patients present with unilateral sensorineural hearing loss (94%) and tinnitus (83%) (Goldbrunner et al., 2020). An inner ear MRI can detect vestibular Schwannoma, large vestibular aqueduct, hemorrhage, and inner ear malformations. In this study, there were no significant differences between the hemorrhage and non-hemorrhage groups (105 vs. 83). Min et al. (2020) reported labyrinthine signal abnormalities in postcontrast heavily T2-weighed three-dimensional fluid-attenuated inversion recovery imaging (HF sequence) in 37.7% of patients with SSNHL and severe-to-profound hearing loss. Increased signal intensity ratio indicates more severe hearing loss and poor prognosis (Min et al., 2020), and Wei et al. (2019) reported that a higher percentage of patients with profound SSNHL induced by inner ear hemorrhage experienced vertigo and had a poor prognosis. In this study, although all enrolled patients had total hearing loss, the results of the inner ear MRI did not always show hemorrhage, and 35 patients had normal results.

There was no statistical difference in sex, affected side, and tinnitus; a result matching those of previous studies (Ceylan et al., 2007). A significant correlation was found between hearing improvement and vertigo and comorbidities. There were 162 (86%) patients with vertigo. Vertigo is known to be a negative prognostic factor (Guan et al., 2020). Simultaneous vestibular and cochlear impairment may be more serious in patients with vertigo than in those without vertigo. Serious vertigo and a sudden total hearing loss were the main reason for the emergent presentation to the hospital, and the time from onset to the start of treatment was less than or equal to 3 days. Therefore, in this study, we did not quantify the time from symptom onset to the start of treatment.

In this study, the subjects’ mean 1 month posttreatment 5-pure-tone hearing thresholds showed poor improvement in general (Figure 3). One (0.5%) 22-year-old man showed complete recovery, whereas 4 patients (2%) had a mean hearing threshold less than 50 dB. None of the groups’ average PTAs improved to less than 50 dB. Wei et al. (2019) reported that the hearing threshold of patients in the non-inner ear hemorrhage group continuously improved from 14 days of treatment to 3 months posttreatment; after which the curve stabilized showing no marked changes across the 3-, 6-, and 12-month follow-ups. In contrast, in the inner ear hemorrhage group, the hearing threshold of the patients improved from 14 days of treatment to 1 month posttreatment, which stabilizes thereafter and shows no obvious changes across the 1-, 3-, 6-, and 12-month follow-ups. In our study, the incidence of inner ear hemorrhage was 56% (108/188), whereas that of non-inner ear hemorrhage was 43% (80/188). Figure 3 shows the average PTA profile in patients with a sudden hearing loss with an initial non-responsive audiogram (except ≥ 71 y, *n* = 8). Considering the hearing improvement and the cost and adverse effects of the drugs used, these average PTAs indicate that more medication and longer treatment duration should not be applied to patients with an initial no-response audiogram after more than 2 weeks of treatment in the hospital.

The mean hearing thresholds showed better improvement in the 18–30-years group than in the other five groups (Figure 2 and Tables 3, 5). This result could be due to a series of factors, which include age-related degeneration, genetic factors, noise exposure, and diseases of the ear and other systems (hypertension, diabetes, dyslipidemia, thyroid disorder, etc.) (Liu and Yan, 2007; Keithley, 2020). Although the hearing level before onset was the same, the physiology of the inner ear varies at different ages. The 50–61-years group had 59 patients and was the largest group in our study. This may be because patients in the transition period to old age are more sensitive to pathogenic factors. First, endothelial dysfunction might play an important role in the pathophysiology of hearing loss through microvascular disturbances and cochlear inflammatory processes (Ciccone et al., 2012). Individuals aged 40 or more than 40 years have increased cardiovascular risk factors and endothelial dysfunction, and this underscores the importance of carefully evaluating the cardiovascular health of patients affected by ISSHL. Second, asymmetrical alterations in venous extracranial hemodynamics may contribute to ISSHL (Ciccone et al., 2018). Cerebral venous and arterial alterations may contribute to the poor hearing improvement with age. Further research is needed to elucidate the pathophysiology of hearing loss and improvement.

In all patients, 0.25 and 0.5 kHz responses improved more than the 1, 2, and 4 kHz responses (Table 5). According to a previous study (Brundin et al., 1989; Furness et al., 2008), sensory hair cells at the cochlea apex respond to low frequencies sound, whereas those at the basal end respond to high-frequency sound. Apical stereocilia are more than two times the height of basal stereocilia. When inner ear microvascular infarction or viral infection occurs, the recovery of apical hair cells is better than that of the other cells.

Our study had several limitations. First, it was a single-center retrospective study, which could have introduced bias; this bias could be reduced by consistent hearing tests and treatments being performed by the same clinicians. Second, the age group that is 71 or more than 71 y included eight patients only; as the main inclusion criterion was a complete lack of response at the initial pure-tone threshold, we had few patients aged 71 or more than 71 years, and we will continue collecting the data of these patients in our ward. We divided the patients into six groups per 10 years because we wanted to know how many dBHL lead to hearing improvement in different ages. Third, subtotal hearing loss was not set as a control group. We will conduct further research regarding this topic. Fourth, 94 patients were lost to the 1-month follow-up. The patients lost trust and were not monitored continuously because of their poor recovery. Self-testing using smartphones, technology similar to that used for detecting middle ear fluid using smartphones, would be more convenient for patients (Chan et al., 2019). This may improve patient compliance and reduce patient loss during the follow-up.

## Conclusion

We reviewed the treatment outcomes and hearing status of large patient groups that suffered from ISSNHL with initial total hearing loss. Our findings showed that the mean PTAs 1 month posttreatment in different age groups were still severe to profound. Although there was a significant age-related difference with respect to marked recovery 1 month posttreatment, and the 18–30-years group showed better recovery than the other age groups; considering the hearing improvement and the cost and adverse effects of the drugs used, these average PTAs suggest that more medication and longer treatment duration should not be used for patients with an initial no-response audiogram.

## Data Availability Statement

The raw data supporting the conclusions of this article will be made available by the authors, without undue reservation.

## Ethics Statement

Ethical review and approval was not required for the study on human participants in accordance with the local legislation and institutional requirements. Written informed consent from the patients/participants or patients/participants legal guardian/next of kin was not required to participate in this study in accordance with the national legislation and the institutional requirements.

## Author Contributions

WX: conceptualization and writing original draft preparation. QD: statistical analysis. YW, ZH, KL, XS, and FD: data collection. HW: conceptualization and funding acquisition. DZ: conceptualization, supervision, writing and reviewing. MW: conceptualization, supervision, writing, reviewing, and editing. All authors contributed to the article and approved the submitted version.

## Conflict of Interest

The authors declare that the research was conducted in the absence of any commercial or financial relationships that could be construed as a potential conflict of interest.

## Publisher’s Note

All claims expressed in this article are solely those of the authors and do not necessarily represent those of their affiliated organizations, or those of the publisher, the editors and the reviewers. Any product that may be evaluated in this article, or claim that may be made by its manufacturer, is not guaranteed or endorsed by the publisher.

## References

[B1] Alexander T. H. Harris J. P. (2013). Incidence of sudden sensorineural hearing loss. *Otol. Neurotol.* 34 1586–1589. 10.1097/MAO.0000000000000222 24232060

[B2] Brundin L. Flock A. Canlon B. (1989). Sound-induced motility of isolated cochlear outer hair cells is frequency-specific. *Nature* 342 814–816. 10.1038/342814a0 2601740

[B3] Ceylan A. Celenk F. Kemaloğlu Y. K. Bayazit Y. A. Göksu N. Ozbilen S. (2007). Impact of prognostic factors on recovery from sudden hearing loss. *J. Laryngol. Otol.* 121 1035–1040. 10.1017/S0022215107005683 17241495

[B4] Chan J. Raju S. Nandakumar R. Bly R. Gollakota S. (2019). Detecting middle ear fluid using smartphones. *Sci. Transl. Med.* 11:eaav1102. 10.1126/scitranslmed.aav1102 31092691

[B5] Chandrasekhar S. S. Tsai, Do B. S. Schwartz S. R. Bontempo L. J. Faucett E. A. (2019). Clinical practice guideline: Sudden hearing loss (update). *Otolaryngol. Head Neck Surg.* 161 S1–S45. 10.1177/0194599819859885 31369359

[B6] Chung J. H. Cho S. H. Jeong J. H. Park C. W. Lee S. H. (2015). Multivariate analysis of prognostic factors for idiopathic sudden sensorineural hearing loss in children. *Laryngoscope* 125 2209–2215. 10.1002/lary.25196 25689850

[B7] Ciccone M. M. Cortese F. Pinto M. Di Teo C. Fornarelli F. Gesualdo M. (2012). Endothelial function and cardiovascular risk in patients with idiopathic sudden sensorineural hearing loss. *Atherosclerosis* 225 511–516. 10.1016/j.atherosclerosis.2012.10.024 23102449

[B8] Ciccone M. M. Scicchitano P. Gesualdo M. Cortese F. Zito A. Manca F. (2018). Idiopathic sudden sensorineural hearing loss and Ménière syndrome: The role of cerebral venous drainage. *Clin. Otolaryngol.* 43 230–239. 10.1111/coa.12947 28744995

[B9] Fontenot T. E. Giardina C. K. Dillon M. Rooth M. A. Teagle H. F. Park L. R. (2019). Residual cochlear function in adults and children receiving cochlear implants: Correlations with speech perception outcomes. *Ear Hear.* 40 577–591. 10.1097/AUD.0000000000000630 30169463 PMC6533622

[B10] Furness D. N. Mahendrasingam S. Ohashi M. Fettiplace R. Hackney C. M. (2008). The dimensions and composition of stereociliary rootlets in mammalian cochlear hair cells: Comparison between high- and low-frequency cells and evidence for a connection to the lateral membrane. *J. Neurosci.* 28 6342–6353. 10.1523/JNEUROSCI.1154-08.2008 18562604 PMC2989617

[B11] Goldbrunner R. Weller M. Regis J. Lund-Johansen M. Stavrinou P. Reuss D. (2020). EANO guideline on the diagnosis and treatment of vestibular schwannoma. *Neuro Oncol.* 22 31–45. 10.1093/neuonc/noz153 31504802 PMC6954440

[B12] Guan R. Zhao Z. Guo X. Sun J. (2020). The semicircular canal function tests contribute to identifying unilateral idiopathic sudden sensorineural hearing loss with vertigo. *Am. J. Otolaryngol.* 41 102461. 10.1016/j.amjoto.2020.102461 32201018

[B13] Hong S. M. Ko Y. G. Park C. H. Lee J. H. Kim J. H. (2012). Analysis of hearing improvement in patients with severe to profound sudden sensorineural hearing loss according to the level of pure tone hearing threshold. *Eur. Arch. Otorhinolaryngol.* 269 2057–2060. 10.1007/s00405-011-1864-8 22143582

[B14] Kanzaki J. (1999). Sudden deafness. *Otorhinolaryngol. Nova* 9 198–202. 10.1159/000027908

[B15] Keithley E. M. (2020). Pathology and mechanisms of cochlear aging. *J. Neurosci. Res.* 98 1674–1684. 10.1002/jnr.24439 31066107 PMC7496655

[B16] Kim J. Jeong J. Ha R. Sunwoo W. (2020). Heparin therapy as adjuvant treatment for profound idiopathic sudden sensorineural hearing loss. *Laryngoscope* 130 1310–1315. 10.1002/lary.28231 31397902

[B17] Liu X. Z. Yan D. (2007). Ageing and hearing loss. *J. Pathol.* 211 188–197. 10.1002/path.2102 17200945

[B18] Lv L. Gao Z. Liu J. Zhuang Y. Hou J. Zhu W. (2022). Comparison between postauricular steroid injection and intratympanic steroid perfusion for refractory severe and profound sudden sensorineural hearing loss. *Am. J. Otolaryngol.* 43:103189. 10.1016/j.amjoto.2021.103189 34492426

[B19] Mattox D. E. Simmons F. B. (1977). Natural history of sudden sensorineural hearing loss. *Ann. Otol. Rhinol. Laryngol.* 86 463–480. 10.1177/000348947708600406 889223

[B20] Min X. Gu H. Zhang Y. Li K. Pan Z. Jiang T. (2020). Clinical value of abnormal MRI findings in patients with unilateral sudden sensorineural hearing loss. *Diagn. Interv. Radiol.* 26 429–436. 10.5152/dir.2020.19229 32755877 PMC7490027

[B21] Thomas J. P. Drewermann S. Voelter C. Dazert S. (2018). Prognostic factors regarding the hearing outcome in severe to profound sudden sensorineural hearing loss treated by tympanotomy and sealing of labyrinthine windows after ineffective systemic corticosteroid application. *Eur. Arch. Otorhinolaryngol.* 275 1749–1758. 10.1007/s00405-018-5023-3 29855690

[B22] Wei F. Q. Wen L. Chen K. Liu M. Wu X. (2019). Different prognoses in patients with profound sudden sensorineural hearing loss. *Acta Oto Laryngol.* 139 598–603. 10.1080/00016489.2019.1605195 31050574

